# Virtual reality and traditional training in surgical instrumentation: A non-inferiority comparative study

**DOI:** 10.1016/j.clinsp.2025.100609

**Published:** 2025-03-21

**Authors:** Cristina Pires Camargo, Sergio Henrique Bastos Damous, Jocielle Santos de Miranda, Patricia Zen Tempski, Francisco de Salles Collet e Silva, José Maria Soares, Edivaldo Massazo Utiyama, Rolf Gemperli

**Affiliations:** a Microsurgery and Plastic Surgery Laboratory (LIM-04), Faculdade de Medicina da Universidade de São Paulo, São Paulo, SP, Brazil; b General Surgery and Trauma Division, Surgery Department, Hospital das Clínicas da Faculdade de Medicina da Universidade de São Paulo, São Paulo, SP, Brazil; c Center for Development of Medical Education, Faculdade de Medicina da Universidade de São Paulo, São Paulo, Brazil; d Department of Endocrine Gynecology and Menopause, Discipline of Gynecology and Obstetrics, Laboratório de Investigação em Ginecologia Molecular e Estrutural (LIM-58), Hospital das Clínicas da Faculdade de Medicina da Universidade de São Paulo, São Paulo SP, Brazil

**Keywords:** Surgical training, Virtual reality, Surgical instrumentation, Education, Technology

## Abstract

**Objective:**

Medical education is undergoing a profound transformation due to technological advancements. Integrating cutting-edge tools like virtual environments and computer-based simulators heralds a new era in teaching methodologies. The objective of the study is to analyze the performance and satisfaction levels of residents undergoing basic surgical skills training using virtual reality.

**Methods:**

The authors recruited residents from the first year in general surgery and allocated them randomly into three groups: a) Text; b) Practice and c) Virtual reality. Initially, the residents completed a pretest for an evaluation and then they were trained according to the group they were assigned to. All the residents performed a 30-minute training session. After that, they took a posttest. Both tests were composed of multiple-choice ten questions. At the end of this study, the authors determined the score by calculating the delta of the performance (Δ = posttest –pretest). After the training, all the participants filled in a five-question satisfaction questionnaire.

**Results:**

Regarding the difference between the pretest and post-test, there was no difference between groups. Only one participant (1/8) showed nausea in the virtual groups. The recommendation and satisfaction after the task were higher in the Virtual reality group compared to the Text group. There was an association between satisfaction after the exercises (VR) and recommendation score.

**Conclusions:**

Virtual reality is safe and non-inferior to traditional surgical instrumentation training. This tool might be a practical and attractive alternative to the traditional instrumentation classes.

## Highlights

• A newly developed tool, featuring a virtual reality immersion headset combined with software created in collaboration with a Brazilian company, enables students to immerse themselves in a simulated surgical theater environment.
• The study demonstrated that virtual reality learning was equivalent to textbook training and scored higher than traditional training in satisfaction ratings.
• The use of virtual reality offers a practical and appealing alternative to conventional instrumentation classes.

## Introduction

In the evolution of surgical training, there has been a marked shift from the historical “Halsteadian” model to a more systematically structured approach.^1^ This contemporary model, anchored in the Muller pyramid for performance evaluation, has redefined how participants' competencies are assessed.^2^, ^3^, ^4^

Crucial and specific academic skill developments are necessary for preparing aspiring surgeons for proficiency in surgical instrumentation. Traditionally, this skill has been acquired through textbook study and instrument analysis since the first years of medical school (clinical practice).^5^^,^^6^

The landscape of medical education is undergoing a profound transformation due to technological advancements. However, the integration of cutting-edge tools like virtual environments and computer-based simulators heralds a new era in teaching methodologies.^7^ In fact, there is a discussion of the value of virtual reality and new technologies. Also, modern technology offers an optimal setting for honing medical and surgical skills.^7^

In response to this changing paradigm, the authors have developed a groundbreaking tool ‒ a virtual reality immersion headset, termed Goggle coupled with a new software we developed in partnership with the company MedxVR (Maceió, Brazil) that allows residents to immerse themselves in a simulated surgical theater environment. This study provided an opportunity to analyze the performance and satisfaction levels of students undergoing basic surgical skills training using this innovative technology, compared to traditional learning methods.

## Methods

### Ethics

This study was approved by IRB (CAAE 61,310,022.1.0000.0068) of the Medical School Hospital of the University of São Paulo and followed Consort guidelines and Brazilian scientific law regulations (RDC 466/2016). Informed consent was obtained from all participants included in the study.

### Software development

The authors developed the surgical instrument module in partnership with the company MedxVR Surgical Instruments (Maceió, Brazil). This software utilizes virtual reality (VR) technology, using Oculus PiCO2, a product of Facebook (Menlo Park, CA).

Virtual reality builds an immersive environment in several scenarios. The first one was a theater; this scenario offers a slide presentation. Theoretical tests were given to the participants. The second section was in an Operating Room (OR). In the OR environment, the participant can manipulate the surgical instruments and set an instrument table according to the specialty chosen.

### Participants

This study was a non-inferiority randomized clinical trial with three arms. The inclusion criteria were surgical residents from first-year surgery programs of both genders.

The residents performed the pre-test and then were randomized by block randomization (six blocks of four participants) by the statistical software Stata 18 (Stata Corp, College Station, TX). Also, the authors performed concealed allocation using opaque sealed envelopes. After concealed allocation, the resident filled out a demographic data form. The residents were divided into three groups:

Text: the residents studied the surgical instruments for 30 mins.

Practice: the residents studied the surgical instruments box for 30 mins.

Virtual reality: training in total virtual immersion for 30 mins, using surgical instrumentation software (MedxVR, Maceió, Brazil).

The students allocated to the groups received all necessary instructions. In the Text group, the students received a booklet with the surgical instruments and in the Practice group the students were allowed to explore the instruments box and then the groups performed the post-test questionnaire.

The students allocated to the Virtual reality group received instructions on how to manage the goggles. The wearable goggle navigation system (PiCO2) for surgical navigation is designed to support real-time display of the surgical scene. In this group, a space of 2 m^2^ is required. The software (MedxVR, Maceió, Brazil) was divided into the following activities: the theater mode, where 10‒20 slides set for four categories (exeresis, hemostasis, suture, and special instruments) are shown for 20 mins. Then, the students were immersed in an operating room. In this module, the participant can manipulate the surgical instruments using handles (used by the operator), and it is possible to simulate the surgeon's hands to reach the surgical instruments. The students could click on an instrument and see an explanation regarding the function. Then, the students must set up an instrument table according to their choice in 10 mins. The students were requested to perform the post-test questionnaire.

### Endpoints

#### Pretest and posttest

The tests were compounded by ten multiple-choice questions related to the name and function of the instrument. For example: “Which of the following instruments is not used for hemostasis?”, “What is the name of the forceps used to grip intestinal segments with minimal trauma to them?”. Also, the authors included clinical cases, such as “Male patient, 57 years old, presents with colicky HD pain. Exploratory open laparotomy was indicated. What instruments are used for this approach?”. The tests were graded at the end of this study, and the final score was calculated by the delta between tests (Δ = posttest–pretest).

#### Analysis of participants’ satisfaction

After the training, the participants filled in a five-question questionnaire (Table 1). This questionnaire was based on the Net Promoter Score, using a scale from 0 to 10, where 0 to 6 were considered detractors, 7 or 8 were neutrals and 9 and 10 were considered promoters.^8^

**Table 1:** Participants’ satisfaction questionnaire.

Questions	Rating
How high do you recommend this course to your colleagues?	0 –10
What is you satisfaction level in terms of educational material?	0 –10
Do you consider that the training time was adequate for your learning process?	0 –10
Did the trainers clearly explain the aim of this training?	0 –10
Are you satisfied with the training?	0 –10

#### Analysis of adverse events

During the study, a senior author evaluated the participants for stress and motion sickness symptoms (dizziness, nausea).

### Statistical analysis

The sample size calculation was performed according to the data from the Kryklywy et al. study.^6^ The number of participants was calculated as *n* = 8 participants per group. A 10 % non-inferiority margin was adopted.

The data is presented by mean and standard deviation. The authors used the Kruskal-Wallis test to compare the groups. If the test was significant, the authors applied Dunn's test as a post-hoc test. The variables gender, video game preference, and hours dedicated to video games were tested for association with satisfaction (categorized in 0 if the grade was <7 and 1 if the grade was higher than 7. For this association analysis, the authors used the Exact Test of Fisher. This study considered alpha = 5 % and power = 80 %. All the analyses were performed using the program Stata 18 (Stata Corp, College Station, TX).

## Results

All the participants completed the trial. The demographic data is shown in Table 2.

**Table 2:** Demographic characteristics of participants.

	Text (*n* = 8)	Practice (*n* = 8)	Virtual reality (*n* = 8)
Age (median –IQR)	26 (24.5–26.5)	25 (24–25)	24.5 (24–25.5)
**Gender (%, n/N)**	Female –37.5 %, 3/8	Female –12.5 %, 1/8	Female –25 %, 2/8
	Male –62.5 %, 5/8	Male –87.5 %, 7/8	Male –75 %, 6/8
**Likes videogames**	Yes –62.5 %, 5/8	Yes –787.5 %, 7/8	Yes –62.5 %, 5/8
**Hours of video game (**<**2h/day)**	100 %	100 %	100 %
**Frequency of video game (at least twice a week)**	100 %	100 %	100 %

**Fig. 1:**
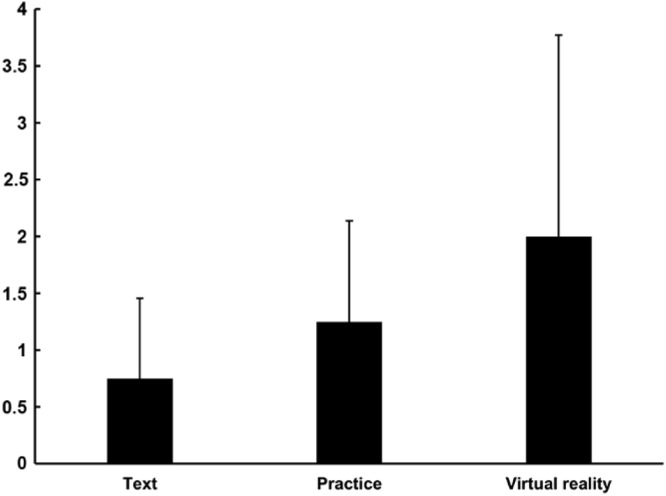
Score determined by the Δ of the performance of the participants (Δ = posttest –pre-test). Data is presented as mean ± standard deviation.

Regarding the difference between pre-test and post-test, there was no difference in all groups: Text, Practice, and Virtual reality (Fig. 1).

**Fig. 2:**
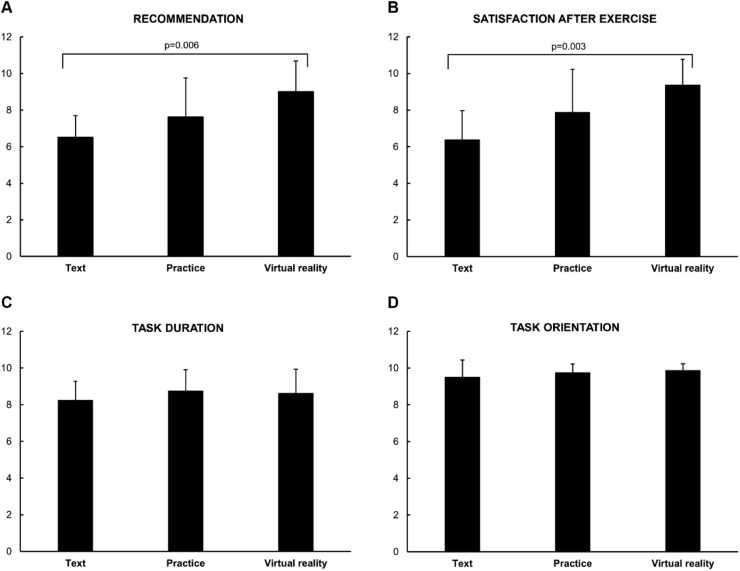
Rating of (A) recommendation, (B) satisfaction, (C) task duration, and (D) task orientation based on the participants’ satisfaction questionnaire. Data is presented as mean ± standard deviation.

Regarding recommendation, the VR group showed higher scores compared to the Text group (Fig. 2A) (6.5 vs. 9.5 respectively, *p* = 0.006). The same outcome was observed in satisfaction after the exercise (Fig. 2B) (6.5 vs. 10, *p* = 0.003).

The perception of adequacy of task duration (Fig. 2C) and task orientation (Fig. 2D) was similar in all groups.

There was an association between satisfaction after the exercises and recommendation score (*p* < 0.001).

### Adverse events

Only one (1/8) participant felt nausea in the virtual reality group.

## Discussion

This study showed that virtual reality learning was equal to textbook training. However, in a systematic review (low level of evidence) about complex surgical training, the authors demonstrated superiority in using virtual reality in educational activities over traditional training.^9^ This difference can happen due to the complexity of the surgical procedures; a high-fidelity model would be more effective than conventional training (low-fidelity procedures). In the present study, the residents had already been trained in surgical instrumentation during graduation.^10^

Regarding satisfaction rating (debriefing, time, recommendation), this study showed higher scores than the other groups.

The authors attribute the high satisfaction scores to the fact that immersion VR has the potential to scale an environment capable of simulating operating room sensory stimuli to the participant. This sensory stimulus, like a video game scenario, increases participant engagement.^11^

Additionally, the authors want to highlight the importance of effective debriefing. In this study, the authors explained all the steps, how to manage the goggles and the joystick, and safety, and showed the common mistakes. Therefore, the time duration and debriefing scores were high (scores bigger than 8). It is essential to explain how to choose a place for this training, as this software demands a physical area to work virtually. In this device, a 2 m^2^ space is necessary to guarantee participants' safety and the effectiveness of the training.

The use of VR in the surgical field reduces training hours, decreasing the workload on the residents’ daily practices. The participant can train in a friendly environment, self-paced, without supervision, and in diverse places.^12^

Another outcome showed a good performance in virtual reality despite gender and interest in video games or practice hours. These findings were different from a systematic review of video game practice and virtual reality.^13^

Regarding adverse events, the trainer could explain some basic movements to avoid any symptoms of motion sickness. To illustrate it, the trainer explains how to move the body and head slowly and smoothly, adjust the goggles, and use glasses if necessary.

In this study, the immersive virtual reality showed low-cost, portable, commercially available hardware. These characteristics are crucial for introducing technology in surgical education.^14^

Although this study did not analyze patient safety, the literature recommends the use of high-fidelity models to ensure patient safety.^15^

## Conclusions

Virtual reality is safe and non-inferior to traditional surgical instrumentation training and might be a practical and attractive alternative to traditional instrumentation classes. Randomized clinical trials with a larger number of participants are necessary to analyze the effectiveness of the technology in surgical training.

## CRediT authorship contribution statement

**Cristina Pires Camargo:** Conceptualization, Methodology, Supervision, Writing – review & editing. **Sergio Henrique Bastos Damous:** Investigation, Data curation. **Jocielle Santos de Miranda:** Investigation, Data curation. **Patricia Zen Tempski:** Conceptualization, Investigation, Formal analysis. **Francisco de Salles Collet e Silva:** Supervision, Methodology. **José Maria Soares:** Writing – review & editing, Validation. **Edivaldo Massazo Utiyama:** Methodology, Writing – review & editing, Supervision. **Rolf Gemperli:** Project administration, Writing – review & editing, Conceptualization.

## Declaration of competing interest

The authors declare no conflicts of interest.
